# Accelerating Dynamic Cardiac MR Imaging Using Structured Sparse Representation

**DOI:** 10.1155/2013/160139

**Published:** 2013-12-18

**Authors:** Nian Cai, Shengru Wang, Shasha Zhu, Dong Liang

**Affiliations:** ^1^School of Information Engineering, Guangdong University of Technology, Guangzhou 510006, China; ^2^Paul C. Lauterbur Research Centre for Biomedical Imaging, Shenzhen Institutes of Advanced Technology, Shenzhen 518055, China; ^3^Shenzhen Key Laboratory for MRI, Guangdong, Shenzhen 518055, China

## Abstract

Compressed sensing (CS) has produced promising results on dynamic cardiac MR imaging by exploiting the sparsity in image series. In this paper, we propose a new method to improve the CS reconstruction for dynamic cardiac MRI based on the theory of structured sparse representation. The proposed method user the PCA subdictionaries for adaptive sparse representation and suppresses the sparse coding noise to obtain good reconstructions. An accelerated iterative shrinkage algorithm is used to solve the optimization problem and achieve a fast convergence rate. Experimental results demonstrate that the proposed method improves the reconstruction quality of dynamic cardiac cine MRI over the state-of-the-art CS method.

## 1. Introduction

Dynamic cardiac cine MR imaging aims at simultaneously providing a series of dynamic magnetic resonance image in spatial and temporal domains (*x*-*t* space) at a high frame rate. It usually acquires the *k*-space at each time frame and collects the raw data in the spatial frequency and temporal domain, the so called *k*-*t* space. Therefore, it is necessary to reconstruct each time frame and get a series of dynamic images. However, the relatively low acquisition speed of the dynamic MRI is an important factor to limit its application in clinics. How to accelerate *k*-space sampling for each time frame and reconstruct them without sacrificing spatial resolution is a challenging problem.

In recent years, many advanced techniques [1–10] were proposed to effectively address this issue and can be divided into two categories. One is based on compressed sensing (CS) theory [11, 12] utilizing the sparsity in dynamic images to be reconstructed, and the other is based on the partial separable theory [13] exploiting the low-rank property of images in *x*-*t* space. The application of CS in dynamic MRI has drawn a lot of attention, since this theory demonstrates that the signal can be accurately reconstructed from a small amount of linear undersampled measurements by exploiting the inherent sparsity in signal. For example, Jung et al. [7, 9] uncovered an intriguing link between the compressed sensing and *k*-*t* BLAST/SENSE and proposed the *k*-*t* FOCUSS algorithm to achieve high spatiotemporal resolution in cardiac cine imaging. Liang et al. [5] developed *k*-*t* iterative support detection (*k*-*t* ISD) method to further utilize the detected partial support information besides the sparsity in cardiac cine images.

Recently, image restoration with patch-based sparse representations has attracted a lot of attention. The similarity of works in this topic is seeking for a more appropriate way to sparsify the image patches than conventional fixed transform. One approach is to provide additional information when using fixed transform on patches. For example, Qu et al. [14] presented to provide the sparsest representation for each image patch by estimating geometric directions. In [15], the nonlocal patches with intensity similarity, instead of those from neighbors, are grouped and then transformed by using a 3D Haar wavelet to produce sparser representation. These two patch-based methods exhibited consistent improvements in reconstruction accuracy over conventional CS-MRI methods. Another approach is based on the dictionary learning technique which aims to learn an adaptive basis from image patches and has shown impressive image restoration results [16]. The essential difference between these two approaches is that the latter uses adaptive learned dictionary instead of fixed basis, such as temporal Fourier transform, as the sparsifying transform. Ravishankar and Bresler [17] have applied the dictionary learning technique to static MR image reconstruction and obtained better reconstruction results than the state-of-the-art methods using fixed sparsifying transform. Liu et al. [18] presented to train dictionaries from the patches of the horizontal and vertical gradient images instead of the pixel domain image. The sparser training samples from the gradient images that are already sparsified by gradient operators result in sparser representation. In [19], a two-level Bregman method with dictionary learning updating is developed by applying the outer-level and inner-level Bregman iterative procedures to update the whole image and image patches, respectively. Experimental results on static MR images demonstrate the superiority of the presented algorithm to the state-of-the-art reconstruction methods.

However, dictionary learning based optimization is a large-scale and highly nonconvex problem, which requires high computational complexity. The coherence of the dictionary and the large degree of freedom may become sources of instability and errors. Structured sparse representation model was proposed to reduce the degree of freedom in the estimations and was thus more stable than conventional sparse representation model. The structured learned overcomplete dictionary, composed of a union of bases of principal component analysis (PCA), was widely used in image restoration [20, 21]. Recently, Dong et al. [22] proposed nonlocally centralized sparse representation (NCSR) model for single natural image restoration, specifically, clustering image patches by *K*-means algorithm at first and then learning PCA subdictionary of each cluster to sparsely represent image patches. Finally, the so-called sparse coding noise (SCN) was minimized to improve the performance of sparsity-constrained image restoration. This model has gotten the satisfactory results on image deblurring, image denoising, and image super resolution.

In this work, motivated by the effective representation ability of NCSR, a novel method based on the NCSR model is proposed to accelerate dynamic cardiac MRI applications. The method utilizes structured sparse dictionary learning to adaptively represent image sequence and reduces the error between the sparse coding coefficients learned by such dictionary and true sparse coding. Improvement of the proposed method over the basic CS approach is demonstrated using retrospectively undersampled in vivo cardiac cine MR datasets.

The rest of the paper is organized as follows. In Section 2, the NCSR model is briefly described and a detailed account of structured sparse representation-based dynamic cardiac MR imaging method is provided. We present experimental validation of our method and compare it to previous state-of-the-art method in Section 3. Conclusions are drawn in Section 4.

## 2. Materials and Methods

### 2.1. Nonlocally Centralized Sparse Representation (NCSR)

Image restoration often requires solving an inverse problem. It amounts to estimate original image vector *x* from a vector of measurements *y*; that is, we have

(1) $$\begin{array}{c} y=Hx+v, \end{array}$$

which is obtained through the noninvertible linear degradation operator **H** and is contaminated by the additive noise *v*.

Mathematically, image vector *x* ∈ *C* ^*N*^ can be represented as *x* ≈ Φ*α* under the sparse representation framework, where Φ ∈ *C* ^*N*×*M*^, *N* < *M* is a dictionary, and *α* ∈ *C* ^*M*^ represents the sparse coefficients. The sparse decomposition of *x* can be obtained by solving a convex *l*_1_-minimization problem:

(2) $$\begin{array}{c} \alpha_{x}=\underset{\alpha }{\arg\;\min}\left\{{||x-\Phi \alpha ||}_{2}^{2}+\lambda {||\alpha ||}_{1}\right\}. \end{array}$$

In the scenario of image restoration, to recover *x* from the degraded image, *y* is first sparsely coded with respect to Φ by solving the following optimization problem:

(3) $$\begin{array}{c} \alpha_{y}=\underset{\alpha }{\arg\;\min}\left\{{||y-H\Phi \alpha ||}_{2}^{2}+\lambda {||\alpha ||}_{1}\right\}. \end{array}$$

And then *x* is reconstructed by $\hat{x}=\Phi \alpha_{y}$. In order to achieve an effective image restoration, *α*_*y*_ are expected to be as close as possible to approach the true sparse codes *α*_*x*_ of the original image *x*. Dong et al. defined the sparse coding noise (SCN) as the difference between *α*_*y*_ and *α*_*x*_:

(4) $$\begin{array}{c} v_{\alpha }=\alpha_{y}-\alpha_{x}. \end{array}$$

Thus, the quality of image restoration can be improved by suppressing SCN. However, *α*_*x*_ is unknown so SCN cannot be directly measured. To address this issue, a good estimation *β* of *α*_*x*_ is necessary. There are various ways to obtain an accurate estimate of *α*_*x*_. Dong et al. tried to learn the estimate *β* by computing the weighted average of the sparse codes of nonlocal similar patches.

The NCSR model was proposed as follows:

(5) $$\begin{array}{c} \alpha_{y}=\underset{\alpha }{\arg\;\min}\left\{{||y-H\Phi \alpha ||}_{2}^{2}+\lambda \sum_{i}{||\alpha_{i}-\beta_{i}||}_{1}\right\}, \end{array}$$

where dictionary Φ can be designed as a PCA-based structured sparse dictionary, *α*_*i*_ denotes the sparse coding vector of *i*th image patch on a certain subdictionary, and *λ* is the regularization parameter controlling the tradeoff between data consistency and sparse coding noise. To solve this problem, firstly, the training patches extracted from the given image are clustered into *K* clusters, and a PCA subdictionary is learned for each corresponding cluster. Then one PCA subdictionary is adaptively selected to code a given patch. Finally, an iterative shrinkage algorithm [23] can be used to solve the NCSR objective function in (5).

### 2.2. NCSR-Based Dynamic Cardiac MR Imaging

When the degradation operator **H** is the under-sampled Fourier encoding operator and *y* is the acquired *k*-space data, we can modify the above model to MR image reconstruction. Based on the NCSR model, we propose a new method to reconstruct a time series of dynamic cardiac cine MR images which have high correlations in the spatial- and temporal-domain.

We define a matrix of image series **X** = [*x*_1_,…, *x*_*S*_] ∈ *C* ^*N*×*S*^, whose columns are the image vectors {*x*_*s*_}, *s* = 1,…, *S*. *S* denotes the number of image frames. In order to reconstruct the image series, we propose the following cardiac cine MRI reconstruction model:

(6) $$\begin{array}{c} \underset{\alpha }{\min}{||Y-F_{u_{\Omega }}\Phi \alpha ||}_{2}^{2}+\lambda \sum_{i=1}^{N}{||\alpha_{i}-\beta_{i}||}_{1}, \end{array}$$

where **X** = Φ*α* and **Y** ∈ *C* ^*N*×*S*^ is the acquired *k*-space data matrix, whose columns are the vector form of *k*-space data of images {*x*_*s*_}. Matrix operator **F**_**u**_*Ω*__ performs the undersampled Fourier encoding, and set *Ω* = {1,2,…, *S*} indicates that the undersampling masks are different for each frame to enforce incoherence. For the selection of the dictionary Φ, we adopt the PCA-based structured sparse dictionary like in NCSR. However, the way to get the patches from the image series {*x*_*s*_} is different from NCSR. We first transform the image series {*x*_*s*_} to the image matrix **X** defined above and then regard the transpose vector of each row **x**_*n*_, *n* = 1,…, *N*, of **X** as a patch vector. In other words, we learn the PCA subdictionaries along the temporal dimension to exploit the inherent correlation in dynamic image series. After getting the patches, we use the *K*-means algorithm to partition the patch set into *K* clusters {*C*_1_, *C*_2_,…, *C*_*K*_} and then compute the covariance matrix Σ_*k*_ of each cluster *C*_*k*_. An orthogonal transformation matrix **P**_*k*_ can be obtained by applying PCA to Σ_*k*_. We set **P**_*k*_ as the PCA subdictionary which can constitute the dictionary Φ.

The iterative shrinkage algorithm is used to solve this problem and the final image series can be obtained from the solved sparse coding vector. Specifically, at each iteration, we use the same method as in NCSR to compute *β*_*i*_. For each local patch, the Euclidean distance was used to search for the first *P* (*P* = 13 in our experiments) closest patches. We applied the corresponding subdictionary to these nonlocal similar patches to obtain their sparse codes. *β*_*i*_ was estimated by computing the weighted average of these sparse codes. This nonlocal method can produce accurate enough estimates of true sparse codes. Finally, the following minimization problem can be solved for a given *β*_*i*_:

(7) $$\begin{array}{c} \alpha_{y}=\underset{\alpha }{\arg\;\min}\left\{{||Y-F_{u_{\Omega }}\Phi \alpha ||}_{2}^{2}+\sum_{i}\sum_{j}\lambda \left|\alpha_{i}\left(j\right)-\beta_{i}\left(j\right)\right|\right\}, \end{array}$$

where *α*_*i*_(*j*) and *β*_*i*_(*j*) are the*j*th elements of *α*_*i*_, *β*_*i*_. We adopted the surrogate algorithm in [23] to solve (7). In the (*l* + 1)th iteration, the proposed shrinkage operator for the *j*th elements of *α*_*i*_ is

(8) $$\begin{array}{c} \alpha_{i}^{\left(l+1\right)}\left(j\right)=S_{\tau }\left(v_{i,j}^{\left(l\right)}-\beta_{i}\left(j\right)\right)+\beta_{i}\left(j\right), \end{array}$$

where *S*_*τ*_(·) is the classic soft thresholding operator. *v* ^(*l*)^ = **K** ^*H*^(**Y** − **K** *α* ^(*l*)^)/*c* + *α* ^(*l*)^, where **K** = **F**_**u**_*Ω*__Φ, *τ* = *λ*/*c*, and *c* is a parameter guaranteeing the convexity of the surrogate function (*c* = 1 in our experiments).

Since one drawback of this iterative framework is the slow convergence rate of *O*(1/*n*), we introduce an accelerated method described in [24] to achieve a fast *O*(1/*n* ^2^) convergence rate. In this method, two prior iterates are used to obtain the next updated solution in the soft thresholding framework. The detailed design is described in Algorithm 1 (b.1) and (b.2).

## 3. Experimental Results

The feasibility of the proposed method was validated on two sets of in vivo dynamic cardiac cine data. Informed consent was obtained from the volunteer in accordance with the institutional review board policy. The full k-space data with the size of 256 × 256 × 25 (number of frequency encoding × number of phase encoding × number of frames) of the first dataset was acquired using a steady-state free precession (SSFP) sequence on a 1.5 T Philips scanner. The flip angle was 50 degrees and TE/TR = 1.7/3.45 msec. The field of view (FOV) was 345 mm × 270 mm and the slice thickness was 10 mm. Retrospective cardiac gating was used with a heart rate of 66 bpm. The second dataset was acquired on a 3 T Siemens Trio scanner (Siemens Medical Solutions, Erlangen, Germany) with a flip angle of 44 degrees and TE/TR = 42.5/1.22 msec. The fully acquired *k*-*t* measurements were of size 304 × 165 × 26 × 12. The FOV was 340 mm × 276 mm and the slice thickness was 6 mm.

The image series reconstructed from the full *k*-*t* data was used as the reference for comparison, while, for the dataset acquired using multiple coils, the image from each channel was reconstructed from full samples and combined using square root of sum-of-squares (SOS) as the reference. To simulate the undersampled *k*-space data, the sampling masks corresponding to reduction factors, *R* = 3 and 4, were generated using the function provided in the *k*-*t* FOCUSS toolbox, where the central 8 phase encoding lines were fully sampled. The fully sampled data were then retrospectively undersampled using the designed sampling masks.

The proposed NCSR-based method and *k*-*t* FOCUSS were used to reconstruct the desired image series with the same sampling patterns for a given undersampled dataset. All methods were implemented in Matlab and the code for *k*-*t* FOCUSS was obtained from http://bisp.kaist.ac.kr/ktFOCUSS.htm. Simulations run on a dual core 2.6 GHz CPU laptop with 4 GB RAM. The running time of our program is about 30 minutes. This time is relatively long due to slow speed in dictionary learning and *K*-means clustering and can be reduced by optimizing the code and utilizing GPU for acceleration.

**Figure 1:**
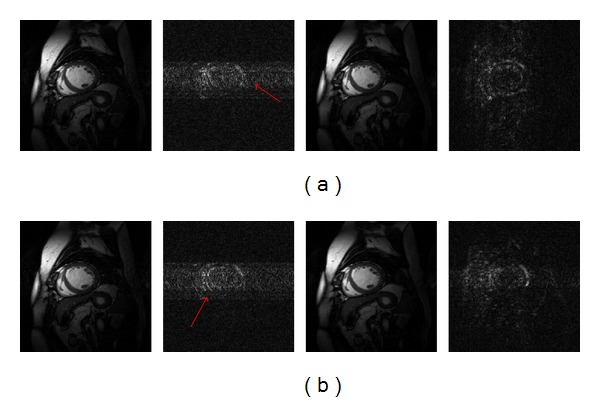
Experiment results in (a) *R* = 3 and (b) *R* = 4. Reconstructions at the 4th frame using *k*-*t* FOCUSS (the 1st column) and proposed method (the 3rd column) and their corresponding difference images (2nd and 4th columns).

**Figure 2:**
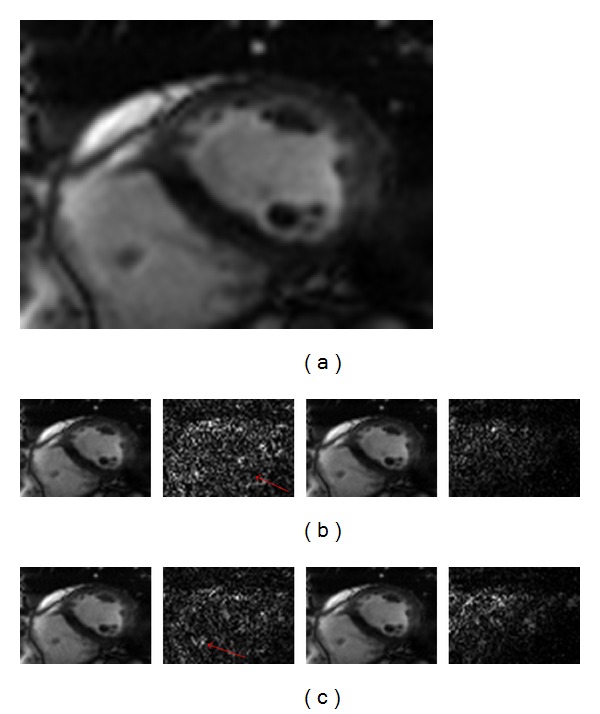
Experiment results of the second dataset in (b) *R* = 3 and (c) *R* = 4. Reconstructions at the 15th frame using *k*-*t* FOCUSS (the 1st column) and proposed method (the 3rd column) and their corresponding difference images. (a) is the full FOV and ROI reference.

The reconstructions with reduction factors of *R* = 3 and 4 and the corresponding difference images at the fourth frame of the first dynamic cardiac dataset are shown in Figures 1(a) and 1(b), respectively. In Figure 1, the first row shows the results of *k*-*t* FOCUSS (the left two) and proposed method (the right two) in *R* = 3, and the second row is the results of two methods in *R* = 4. It can be seen from the reconstructed images that the *k*-*t* FOCUSS presents more undersampling artifacts along the phase encoding direction. Our method greatly suppresses the artifacts and obtains high quality reconstructions. The superiority of structured sparse representation based method is also clearly seen in the difference images as shown by the red arrows. Figures 2(b) and 2(c) show the region-of-interest (ROI) reconstructions using *k*-*t* FOCUSS and the proposed method (from left to right) with *R* = 3 and 4 (from top to left) of the second dynamic cardiac dataset. We can find some artifacts appearing in the *k*-*t* FOCUSS result especially with a reduction factor of 4.

**Figure 3:**
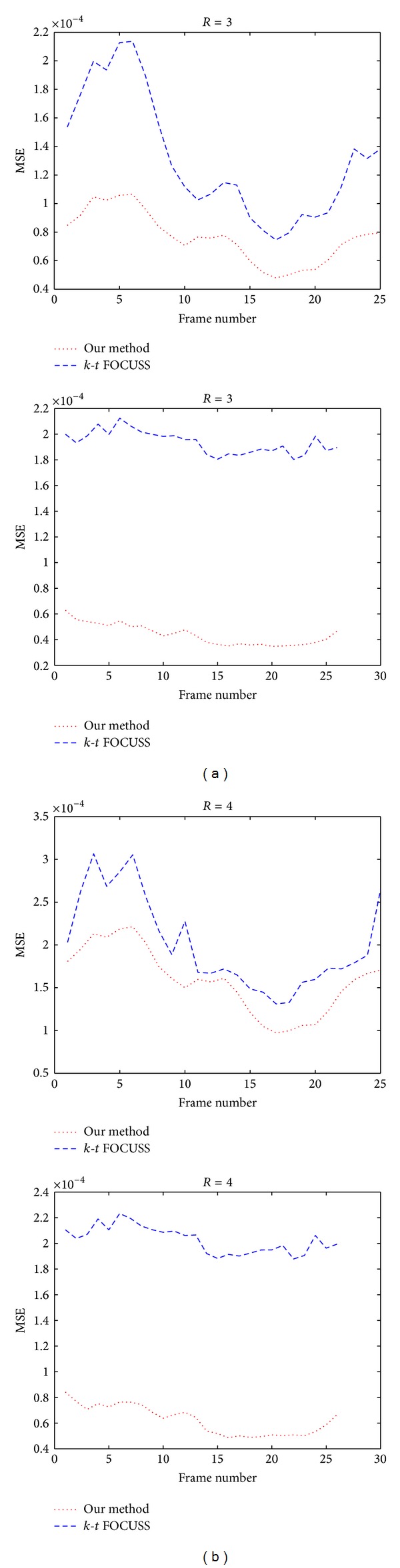
Frame-by-frame plots of NMSE for *k*-*t* FOCUSS and our method with (a) *R* = 3 and (b) *R* = 4 in the first dataset (the 1st row) and the second dataset (the 2nd row). The dotted lines are for our method and dashed lines for *k*-*t* FOCUSS.

To quantify the improvement of the proposed method over *k*-*t* FOCUSS, the normalized mean-squared error (NMSE) between the reconstruction and the reference at *R* = 3 and 4 was calculated and plotted as a function of time frame in Figures 3(a) and 3(b), respectively. The nRMSE was calculated using the following formula:

(9) $$\begin{array}{c} \text{nRMSE}=\sqrt{\frac{\sum_{i=1}^{N}{\left(x_{\text{rec}}\left(i\right)-x\left(i\right)\right)}^{2}}{\sum_{i=1}^{N}x{\left(i\right)}^{2}},} \end{array}$$

where **x**_rec_ is the reconstructed images from the undersampled data, **x** is the reference, and *N* is the image size. The dotted lines are for our method and dashed lines for *k*-*t* FOCUSS, respectively. Our method is seen to have a lower MSE than *k*-*t* FOCUSS for all frames at specified reduction factors.

**Figure 4:**
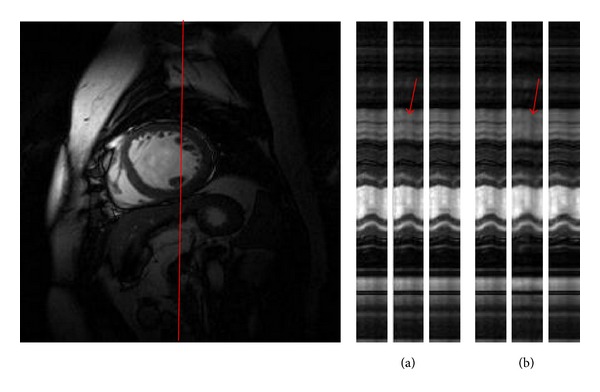
The temporal profiles in *x*-*t* plane of different reconstruction methods with (a) *R* = 3 and (b) *R* = 4 in the first experiment. The results are from reference (left), *k*-*t* FOCUSS (middle), and our method (right) for each reduction factor.

The ability to catch the dynamic motion along temporal direction is a key factor for comparing different dynamic reconstruction methods. To evaluate the temporal fidelity, we show in Figure 4 the reconstructions in *x*-*t* plane of the first dataset with *R* = 3 and 4 for a fixed position in the frequency-encoding direction. It is seen that *k*-*t* FOCUSS shows some loss of contrast. In comparison, the proposed method preserves more temporal variations especially in regions indicated by red arrows.

**Figure 5:**
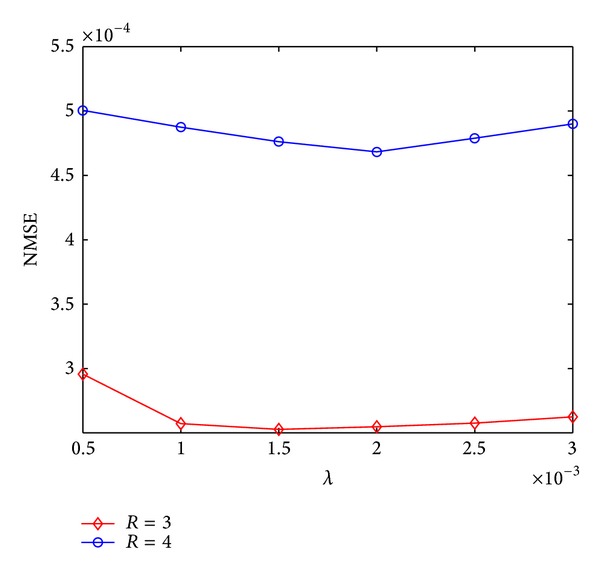
NMSE of 10th frame of the first dynamic cardiac dataset versus regularization parameter *λ* with *R* = 3 and 4.

In our algorithm, a regularization parameter *λ* was introduced. This parameter controls the tradeoff between the data fidelity and the accuracy of the sparse codes, and it also affects the thresholds *τ* in (8). In this work, *λ* was elaborately tuned in a parameter range. To show the effects of this parameter on final reconstructions, the curves of NMSE with respect to parameter *λ* for the 10th frame of the first dynamic cardiac dataset at *R* = 3 and 4 were plotted in Figure 5. We can find that the reconstructions are relatively robust to this parameter. Results with least NMSE are obtained when *λ* = 0.0015 with *R* = 3 and *λ* = 0.002 with *R* = 4. In our experiments, we empirically set *λ* = 0.002.

**Figure 6:**
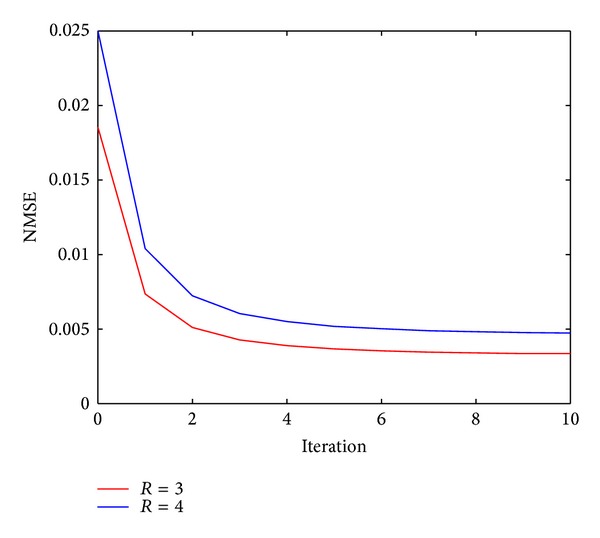
NMSE curves of the first dynamic cardiac dataset versus outer iteration number with *R* = 3 and 4.

The convergence behavior is an important factor in evaluating the performance of the proposed method. The corresponding NMSE-iteration plots are shown in Figure 6 when *R* = 3 and 4 for the first dataset. It can be seen that the NMSE decreases fast at the first few iterations and then becomes flatter and reaches the convergence zone after 6 outer iterations.

From the above experimental results, we can find that our method produces more accurate reconstruction on image sequence than *k*-*t* FOCUSS. It is because we force the sparse coefficients of dictionary learning to approach the true sparse coding, which is estimated through the nonlocal similarity technique. This technique was proved to be an effective method using image redundancy and therefore the accurate sparse representation promotes the quality of reconstruction.

## 4. Conclusions

In this work, we propose a novel dynamic cardiac MR imaging method based on the NCSR model. This method sparsely codes the image sequence by adaptively learning PCA-based structured sparse dictionary and recovers the true sparse coding coefficients with a centralized sparse constraint, which effectively exploits the image nonlocal redundancy. An accelerated iterative shrinkage method was presented for solving the proposed model. From the experimental results from in vivo dynamic cardiac cine MR imaging, it is proved that the proposed method could produce fewer artifacts and preserve contrast than the state-of-the-art method.

## Figures and Tables

**Algorithm 1:**
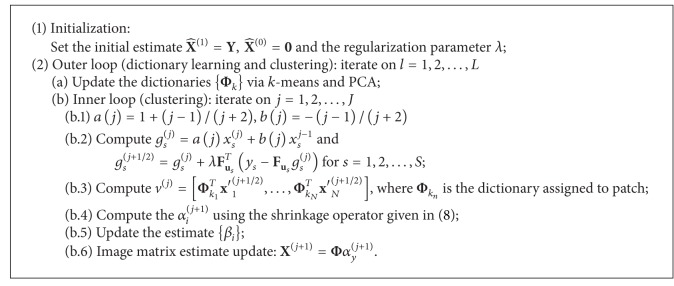
NCSR-based dynamic cardiac cine MR imaging.
